# A Visit to Foreign Dental Schools and Other Observations

**Published:** 1886-11

**Authors:** A. W. Harlan

**Affiliations:** Chicago, Ill.


### A VISIT TO FOREIGN DENTAL SCHOOLS AND OTHER OBSERVATIONS.

BY A. W. HARLAN, M. D., D. D. S., CHICAGO, ILL.

(Continued from page 397.)

Germany, to the American mind, usually includes North and South Germany and Austro-Hungary, having a combined population larger than Great Britain, Ireland, Belgium, Holland, Switzerland and Italy. Within the realms of the German Empire and the Austro-Hungarian monarchy are many comparatively large cities and towns without a single practicing dentist. In 1880 there were cities of ten to twenty thousand population where the only dentist was the local cupper and leecher, who was likewise, in many instances, a tonsorial artist. In consequence of these facts and the low wages of German artisans, laborers, and those employed in agricultural pursuits, we find a poor appreciation of the services of dentists among people who, in the same ranks at home, have their teeth attended to with reasonable regularity. The residents of such towns requiring dental skill, and having the means to employ it, generally go to the large cities of their own country, or to cities of neighboring countries, such as Venice, Milan, Geneva, Turin, Rome, Zurich, Paris, Brussels, Amsterdam, The Hague, Copenhagen, or Stockholm, and occasionally to St. Petersburg, Moscow, etc. Those whom they employ, thereby gain a continental reputation, which is of great value to them at home. When it is known that Count X. or W. employs Dr. G. or K., others from his own locality flock thither. Hence there is little inducement for an educated and competent dentist to locate in a comparatively small city or town, unless it be a health resort, a growing manufacturing city, or the residence of members of the nobility. These, I believe, are some of the reasons why there are so few dentists in many places of a hundred thousand inhabitants or less.

Many things are different in the Old World, when viewed from any standpoint that we may choose as our point of observation. At home curiosity prompts many people to try a new comer in almost any profession, while in the older countries, unless the new arrival is introduced by the late proprietor, or is his copartner, or unless there be some other fortunate circumstance, he may have to wait a long time for a paying practice, and often he will not get it at all. There was a time when almost any American dentist could locate in a foreign country and put up a sign "American Dentist," and soon have all he could well attend to, but those days are past. Too many Germans and others now possess American degrees, and they put up signs of the same character, and issue cards with "*Dentiste Americain*" on them, and take the native into their folds as easy as we can whistle Yankee-doodle. There was a period in the history of dentistry when it was our proud boast that no one could fill a tooth like a native Yankee, or nearly as well as he, but I believe there are many men of all nationalities, spread all over Europe, who are quite our equals as operators. In proportion to the number of practicing dentists, however, we can still take the lead, and I think the State of Illinois has more fine operators than the whole

German Empire could muster, of native material, although some of them can and do make fine fillings, both of soft and cohesive gold. I have seen them, and know whereof I affirm. We attempt to save, and succeed in saving, more teeth than they do, while the German dentist, like his English brother, either extracts, pivots, or fills with cement or amalgam. I speak now of the average every-day dentist, and he easily outnumbers the really skilled operator ten to one. In Germany one sees many front teeth that have been filled with amalgam. This is a practice which American dentists are too conscientious to indulge in to any great extent. It may be that a few unscrupulous men at home practice in this way, but I think such practice is very limited. I will say this, however, for my German *confrère:* he undoubtedly saves more teeth by so practicing than he would by inserting poor gold fillings. I believe the average German dentist makes a better amalgam filling than the dentist of the same grade at home. He takes more time, and the amalgam has more copper in it than that which is used in the United States.

American filling materials and instruments are sold in all the large cities where there are dental depots, so that the American dentist practicing there has as much to select from as we have in New York, or elsewhere. The German oxy-chlorides and oxy-phosphates are equal, if not superior to our own, and everyone now knows how wonderfully soft is the Wolrab gold. The rubber dam is used to a limited extent in the cities, but not nearly so universally as at home. The rubber of German manufacture is of excellent quality, and I saw some that was rough on one side and smooth on the other that I liked very much. It is easy to adjust, as the fingers do not slip in stretching it over the teeth.

In Berlin, as elsewhere, when you ask for novelties they always bring out something from America. Reflectors and lamps for illuminating the mouth, and immense machines for vulcanizing and stamping plates (metallic), pneumatic pluggers, etc., are very common. I noticed that all instruments for filling were larger than ours, with the exception of mouth mirrors. Of these latter I saw many forms, very neat in size and shape. I found some very beautiful broach holders and nerve broaches, also. The silk paper for making cones for drying out root canals and for conveying medicines to the apices of roots is very useful.

Dental fees in Germany are low. I was told that even the best

American dentists seldom received larger sums for a single filling than 60 to 90 marks ($15 to $22), the minimum being from 15 to 20 marks ($3.75 to $4.80). I believe that teeth are filled with amalgams and cements lower than in England, some dentists making such fillings for from 1 mark (24 cts) upwards. The best dentists of course receive higher fees, but they are not so high in Germany as in England. Artificial teeth are ridiculously cheap, as there are many who only extract teeth and make substitutes. There are many good mechanical dentists in the larger cities, but I should say, from my own observation, that they are better mechanicians than artists, as they do not take nature for their models. The teeth made by them look artificial and unnatural. Many plates are made of silver. I saw very few natural looking artificial teeth in the mouths of those I examined. The custom prevails of exhibiting boxes of teeth, filled and unfilled, of sets on all kinds of bases, elixirs, brushes, powders, etc. These are usually at the entrance to the stairway leading to the apartments of the dentist. None of the first-class dentists resort to this method of advertising in any of the old countries. (*En passant,* there is one short street in London where there are located about fifteen or twenty dentists (?) on opposite sides of the street, whose sole stock in trade appears to be exhibited in immense show windows extending across the whole front of the house; each article, from a tooth-pick to a set on gold, is labeled with the price. Signs reading : "Springs put on while you wait," "Sets made complete in six hours," and numerous others quite amusing to read, all conspicuously displayed.) Of course there are many poorly educated men practicing dentistry everywhere, and we cannot boast much of our own advertising dentists, but they do not come quite up to the tactics of some of those in the Old World. Quackery is usually the outgrowth of ignorance in the first instance, but it must be fed by all classes, judging by the testimonials which one reads on the hand-bills of some European dentists, and the pages of pamphlets and newspapers in our country. The millennium will undoubtedly arrive when they are all dead and gone, and their places are filled by the new generation of dentists, whose preliminary education will be such that they will know before entering upon the study of dentistry that hydrated starch when brought into contact with human saliva will *not* produce indigestion and constipation.

Previous to the opening of the Dental Institute in connection with the University of Berlin, systematic study of dentistry was not insisted upon in the German Empire. It is true that in a few cities where Universities were located, such as Breslau, Halle, and one or two others, a certain amount of instruction was given by lecturers from models, and the construction of artificial teeth was taught in the same way; but these courses were not comprehensive, and the certificates issued were not in the shape of a diploma. In order to obtain the title of "Zahnarzt," the candidate after pursuing his studies (where he pleased generally) was examined by a commission, and if found qualified the coveted title was his. Where titles were not issued by a University under its seal, the commission was appointed by the State, so that the possessor of the title could not add after his name and title "Leipzig," "Tuebingen," or the name of any University. I presume the graduates from the Institute in Berlin, and others where dental departments are established by the authorities, will be permitted that privilege in the future. Hence, in a few years, we will read Dr. J. Putgelli, Zahnarzt (Berlin), 20 Brown Street, Cosmopolis, W. T.

I spent a portion of two afternoons in the rooms of the Dental Institute in Berlin. I should say that there were about thirty or thirty-five students engaged at the chairs, either operating or assisting each other. The professors are also the clinical instructors, which is a good feature to begin with. All the lectures on anatomy, physiology and other strictly medical subjects, are taken by the dental students in the medical departments. The students are required to take practical instruction in the infirmary during two semesters of five months each. They are also required to work in the dental laboratory on practical cases for the same length of time. One of the professors told me that the operating and laboratory work was not obligatory in the rooms of the Institute, if the candidate for honors possessed the desired knowledge at his final examination. I presume, in time, the authorities will insist on this. The rooms are well equipped, but the light is not as good as it might be. Like students everywhere, they prefer to make gold fillings. Many of them wore glasses, which is quite common in Germany, in all ranks of life. On account of the necessity for the diploma of a gymnasium (high school or academy), before entering college, the students are older than ours. There were no women in the class. I witnessed some of the operations, and they were quite equal to the average in this country. All the lectures on dental subjects are delivered by three or four professors and one assistant, as are also the demonstrations in the various departments. I was told that there were nearly a hundred matriculates for dental honors in the University. Many of them were pursuing their primary studies in other departments, so I did not see them. I predict a brilliant future for the Institute, on account of the length of the period of study, as it will require about five or six years, on an average, for the student to emerge a full-fledged Zahnarzt. At present the clinic rooms are not in the University buildings, but are located some distance from them, which is an inconvenience, but by and by they expect to have a new building of their own.

The establishment of Dental Institutes in the Universities is a good thing for German dentistry, as they will, in a few years, raise the standard so that one cannot find a dentist who has not used "one-eighth of an ounce of gold in ten years' practice." I doubt not that there are to-day dentists in Berlin with a large practice who do not use one-half ounce of gold in a year. The graduates of the Institutes will change this, and they will also awaken the appreciation of the middle and lower classes by doing operations in their clinics. They do not charge for anything but gold fillings in the Institute, which is a mistake in my opinion, for reasons already stated in the May number of this Journal.

Dental journalism flourishes in a larger degree in Germany than in England, and it will be noticed that there are more journals published in that country. Several of them present original communications in each number, but many times their pages are filled with matter from foreign sources, a practice which we at home do not very strongly approve. If a better system of condensation were employed they could give more professional *news*, and this, it would seem, would be equally valuable for their readers. It may be, however, that the editors know what will suit their readers better than the writer. At one time or another I have seen "The Deutsche Monatsschrift fur Zahnheilkunde," "The Correspondenz Blatt fur Zahnarzte," "Centralblatt fur Zahnheilkunde," "Die Zahntechnische Reform," "Vierteljahrsschrift des Vereins Deutscher Zahnheilkunde," "Oesterreichisch-Ungarische Vierteljahrsschrift fur Zahnheilkunde," and that invaluable publication known as Petermann's "Zahnarztlicher Almanach." If I have omitted any journal in the above list, it is because I have never seen a number of it. If there are no others published in Germany and Austria, it seems to me that this list would convince the unprejudiced observer that German dentists were readers, and when a nation reads we are pretty certain to hear from it, either practically or scientifically.

I have not had the pleasure of attending a real, live German dental society, hence it is not possible for me to say anything about their methods of conducting one. But, from the number now in existence (twelve or fifteen), not counting small congregations in outlying districts, they must have about as lively times as we at home. Their custom is, I believe, to elect men to office who have done meritorious work in some special field of labor, and continue them in office until a worthy successor is found willing to take the reins of government. (At home we do not follow this practice, preferring in general to name a new set of officers each succeeding year.) The German method is, in my opinion, the better and safer one to follow, especially in all delegated bodies. Much original work is presented by the members of German dental societies, but the results of investigations are not so quickly or readily accepted as demonstrated facts by them, as they (the members of societies) are more conservative than Americans.

The American Dental Society of Europe has been, and is now, an element of power for advancement in all foreign countries, and many Germans educated in American schools are members of it, and they are always heartily welcomed at its meetings. Judging from the reports of its meetings, the Germans are doing much in the way of working out practical problems and contributing their share in the march of scientific progress, so ably begun by our own industrious and indefatigable investigator, W. D. Miller.

In closing this disjointed and rambling article on German dentists and dentistry, I have only to say that all is spoken with friendliness and without malice, for I was handsomely treated and entertained in the most hospitable manner, and I hope at some future time to reciprocate the courtesies shown me by native Germans. In my next communication I will pay my respects to *La Belle France et les Français.*

(TO BE CONTINUED.)

